# Interplay of two signals in a neuron with heterogeneous synaptic short-term plasticity

**DOI:** 10.3389/fncom.2013.00086

**Published:** 2013-07-18

**Authors:** Felix Droste, Tilo Schwalger, Benjamin Lindner

**Affiliations:** ^1^Bernstein Center for Computational NeuroscienceBerlin, Germany; ^2^Department of Physics, Humboldt Universität zu BerlinBerlin, Germany

**Keywords:** short-term plasticity, information transmission, information filtering, multi-sensory integration, stochastic resonance

## Abstract

Signals from different sensory modalities may converge on a single neuron. We study theoretically a setup in which one signal is transmitted via facilitating synapses (*F* signal) and another via depressing synapses (*D* signal). When both signals are present, the postsynaptic cell preferentially encodes information about slow components of the *F* signal and fast components of the *D* signal, whereas for a single signal, transmission is broadband. We also show that, in the fluctuation-driven regime, the rate of information transmission may be increased through stochastic resonance (SR). Remarkably, the role of the beneficial noise is played by another signal, which is itself represented in the spike train of the postsynaptic cell.

## 1. Introduction

A single neuron can receive inputs that encode more than one signal. The combined effect of several stimuli on the postsynaptic spike train can be regarded as a non-trivial signal interaction. When stimuli stem from different sensory modalities (like vision and hearing), such interaction is often referred to as multisensory integration (Shimojo and Shams, 2001; Driver and Noesselt, 2008) and has been demonstrated on many levels, ranging from behavioral experiments (Sekuler et al., 1997; Shams et al., 2000) over fMRI (Macaluso et al., 2000) and EEG studies (Giard and Peronnet, 1999) to intracellular measurements in single neurons (Meredith and Stein, 1983; Stein and Stanford, 2008).

Typically, studies of multisensory integration consider the overall increase or decrease in firing rate. The information transmission about each of the *time-dependent* stimuli and how it is affected by the interaction of signals has received less attention. However, temporal features may play an important role in multisensory integration. It thus seems worthwhile to study theoretically how two time-varying signals can interact in a neuron.

In general, signals may differ in their temporal structure and in the way they enter the postsynaptic dynamics, e.g., on different dendritic or somatic locations (Rowland et al., 2007). On a functional level, the synapses transmitting one signal may differ in their filter properties from the synapses transmitting the other. Synaptic filter properties are shaped by short-term synaptic plasticity (Zucker and Regehr, 2002): upon repetitive stimulation, synaptic efficacies can either increase (facilitation) or decrease (depression), depending on the nature of the synapse. This usage-dependence of synaptic transmission endows synapses with a variety of functions relevant to information processing (Abbott and Regehr, 2004).

An important aspect of neural information transmission is whether neurons preferentially transmit information about slow or fast components of a signal, i.e., whether they act as a filter of sensory information (Chacron et al., 2003; Krahe et al., 2008; Middleton et al., 2009; Sharafi et al., 2013). Previous theoretical studies have found that information transmission through homogeneous populations of dynamic synapses does hardly depend on signal frequency (Lindner et al., 2009; Merkel and Lindner, 2010), although it has recently been shown (by taking synaptic stochasticity into account) that this is only true for large synaptic populations (Rosenbaum et al., 2012). Filter properties of heterogeneous populations, in which synapses differ in their dynamics depending on the kind of presynaptic cell, have not yet been studied. Examples for such a scenario include Purkinje cells [parallel/climbing fibers making facilitating/depressing synapses, (Kandel et al., 2000)] and simple cells in cat visual cortex [cortico-cortical facilitating and thalamo-cortical depressing synapses, (Banitt et al., 2007)]. In this paper, we study the neural transmission of two independent signals that enter via such distinct synaptic populations.

Specifically, we explore theoretically how the presence or absence of one signal can influence the neuron's information transmission properties with respect to the other signal. To this end, we use information-theoretic measures to quantify (a) the amount of information transferred about certain frequency components of the signal and (b) the total amount of information transferred about each of the signals. We find that the target neuron preferentially encodes information about slow components of the signal impinging on facilitating synapses and fast components of the signal impinging on depressing synapses when both signals are present. This is in contrast to the case of only one signal, in which information transfer is largely frequency independent. Further, we find that the presence of a second signal can increase the total rate of information transmission about the first signal, through the effect of stochastic resonance (SR) (Gammaitoni et al., 1998). In order to clarify the role of short-term plasticity for SR, we also compare to a setup with static synapses.

## 2. Materials and methods

### 2.1. Model

**Figure 1:**
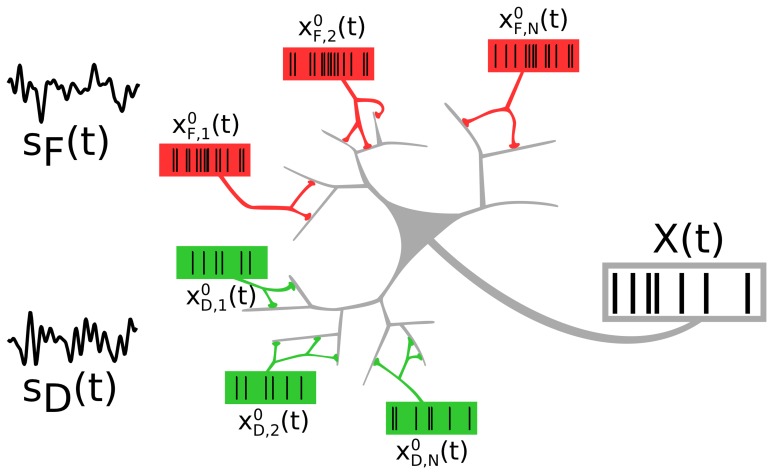
**A neuron receiving spike trains via two types of synapses showing short-term facilitation (red) or depression (green).** Presynaptic populations are assumed to fire Poissonian spike trains, either at a background rate or an elevated rate, modulated by a signal *s*_*F*_(*t*) (“*F* signal,” for the facilitating synapses) and *s*_*D*_(*t*) (“*D* signal,” for the depressing synapses). We address the question of how the two independent signals are represented and how they interact in the output spike train.

We consider a neuron that receives inputs from two distinct neural populations, each of which encodes an independent signal (see Figure 1). The two presynaptic populations differ in the synaptic connections they make onto the target cell: One signal (*F* signal) is encoded in spike trains impinging on facilitating, the other (*D* signal) in spike trains impinging on depressing synapses.

#### 2.1.1. Input

The spiking activity of each presynaptic neuron (*N* = 500 per population) is modeled as an independent Poisson process $x_{P,n}^{0}(t)=\sum_{i}\delta (t-t_{i,n}^{P})$ [where *P* is *F* or *D*], with a common instantaneous rate *R*_*P*_(*t*) for all the neurons within one population. A population *P* can be in a background state, in which all neurons fire at a low constant rate *R*_*P*_(*t*) = *r*_0_ = 1 Hz. Alternatively, the population may be in a signaling state, in which a time-dependent signal *s*_*P*_(*t*) is encoded in the common instantaneous rate *R*_*P*_(*t*) = *r*_*P*_ (1 + ϵ_*P*_ *s*_*P*_(*t*)), with a higher baseline rate *r*_*P*_ = 20 Hz. For the two signals, *s*_*F*_(*t*) and *s*_*D*_(*t*), we use two independent band-limited Gaussian white noise processes, each with unit variance and cutoff frequency *f*_*c*_ = 10 Hz; the modulation amplitude is ϵ_*P*_ = 0.05.

#### 2.1.2. Synapses

We use a deterministic model of synaptic short-term plasticity, close to the models proposed or used in Tsodyks and Markram (1997), Markram et al. (1998), Dittman et al. (2000), Lewis and Maler (2002, 2004), Lindner et al. (2009) and Merkel and Lindner (2010). We use purely facilitating and purely depressing synapses, which is an idealization but allows us to use theoretical expressions developed in Merkel and Lindner (2010). We have verified that using synapses that are *predominantly* facilitating or depressing leads to qualitatively similar results.

Facilitating synapses are governed by

(1) $$F_{n}(t)=F_{0,\;F}+{\left(\frac{1}{1-F_{0,F}}+\frac{1}{F_{C,n}(t)}\right)}^{-1},$$

(2) $${\dot{F}}_{C,\;n}(t)=-\frac{F_{C,n}(t)}{\tau_{F}}+\Delta \cdot x_{F,n}^{0}(t),$$

while depressing synapses obey

(3) $${\dot{D}}_{k}(t)=\frac{F_{0,D}-D_{k}(t)}{\tau_{D}}-F_{0,\;D}D_{k}(t^{-})\cdot x_{D,k}^{0}(t).$$

Here, *F*_*n*_(*t*^−^) (*D*_*k*_(*t*^−^)) is the probability that a *functional contact* of the *n*th facilitating (*k*th depressing) synaptic connection releases a neurotransmitter-filled vesicle upon spike arrival. Under functional contacts, we subsume multiple synaptic boutons, multiple active zones per bouton, or any other physiological feature that allows a synapse to release more than one vesicle onto the target neuron. By the “-”-superscript to the time argument, we denote the evaluation of this variable immediately *before* it is itself influenced by the incoming spike. Between spikes, release probability relaxes to its intrinsic value *F*_0, *F*_ = 0.05 (*F*_0, *D*_ = 0.4) on a time scale τ_*F*_ = τ_*D*_ = 50 ms; we set Δ = 0.175.

As is commonly done in this kind of deterministic model, we approximate the effect of a presynaptic spike on the postsynaptic conductance by considering the trial average of the number of vesicles released. This means that the jump in postsynaptic conductance induced by a presynaptic spike at the *n*th facilitating (*k*th depressing) synapse is *aF*_*n*_(*t*) (*aD*_*k*_(*t*)), where *a* = 10 nS is the jump in conductivity if all functional contacts release their vesicles. This corresponds roughly to 10 functional contacts that each presynaptic neuron makes onto the target cell.

In reality, release of synaptic vesicles is probabilistic, and once a vesicle has been released, additional variability is introduced by the stochastic nature of vesicle recovery. Synaptic depression then emerges naturally as a consequence of resource depletion. As it has recently been shown that synaptic stochasticity can influence the filtering of rate coded information (Rosenbaum et al., 2012), we have verified that the effects we describe below are also found in simulations with stochastic synapses. For these simulations, we have used a stochastic model for depression dynamics (Vere-Jones, 1966; Fuhrmann et al., 2002; Loebel et al., 2009; Rosenbaum et al., 2012), combined with the deterministic facilitation dynamics [Equation (1), (Dittman et al., 2000; Lewis and Maler, 2002, 2004; Merkel and Lindner, 2010)]: Upon spike arrival, each functional contact with a release-ready vesicle at the *n*th facilitating (depressing) synapse independently releases its vesicle with probability *F*_*n*(*t*^−^) (*F*_0, *D*_). The jump in postsynaptic conductance induced by vesicle release is *aN*_*n*, *R*_(*t*)/*N*_*C*_, where *N*_*n*, *R*_(*t*)_ is the number of vesicles released and *N*_*C*_ is the number of functional contacts. In depressing synapses, used vesicles get replaced after exponentially distributed waiting times with time constant τ_*D*_, while in purely facilitating synapses, we model vesicle replacement as instantaneous. For *N*_*C*_ → ∞, we recover the deterministic model Equation (3).

#### 2.1.3. Target cell

The dynamics of the total postsynaptic conductance is given by

(4) $${\dot{g}}_{e}(t)=-\frac{g_{e}(t)}{\tau_{e}}+a\;X_{\text{in}}(t),$$

where the total input

(5) $$X_{\text{in}}(t)=\sum_{n}^{N}x_{F,\;n}(t)+\sum_{k}^{N}x_{D,\;k}(t)$$

is the sum over all incoming spike trains weighted by facilitating and depressing synaptic dynamics,

(6) $$x_{F,\;n}(t)=F_{n}(t^{-})x_{F,\;n}^{0}(t),\;x_{D,k}(t)=D_{k}(t^{-})x_{D,k}^{0}(t).$$

The target cell is a leaky integrate-and-fire neuron

(7) $$C\dot{V}(t)=-g_{L}(V(t)-E_{L})-g_{e}(t)(V(t)-E_{e})+I_{i},$$

where *C* = 300 pF is the membrane capacitance, *g*_*L*_ = 15 nS the leak conductance, *E*_*L*_ = −60 mV the leak reversal potential, and *E*_*e*_ = 0 mV the excitatory reversal potential. We approximate local inhibition by a constant current *I*_*i*_, allowing us to control whether the neuron is in a supra-threshold or a sub-threshold regime. When *V* reaches a threshold *V*_*th*_ = −50 mV, the neuron emits a spike and the voltage is reset to *V*_*r*_ = −62.5 mV. We define the output spike train as $X(t)=\sum_{i}\delta (t-t_{i}^{*})$, where *t*_i_^*^ is the time of the *i*th threshold crossing.

### 2.2. Measure of information transmission

In order to assess how the signals *s*_*F*_(*t*) and *s*_*D*_(*t*) are encoded in the output spike train *X*(*t*), we utilize spectral measures. In simulations, we use a finite-time-window version of the Fourier transform, which for a time series *x*(*t*) is given by

(8) $${\tilde{x}}_{T}(f)=\int_{0}^{T}dt\;e^{2\pi ift}x(t),$$

while in analytical calculations, it is advantageous to use the infinite-time-window transform

(9) $$\tilde{x}(f)=\int_{-\infty }^{\infty }dt\;e^{2\pi ift}x(t).$$

Denoting trial averaging by brackets and complex conjugation by an asterisk, we approximate the cross- and power spectra of two time series *x*(*t*) and *y*(*t*) (where *x* = *y* for power spectra) as

(10) $$S_{xy}(f)=\frac{1}{T}\;\langle {\tilde{x}}_{T}(f){\tilde{y}}_{T}^{*}(f^{\prime })\rangle ,$$

for simulations (according to the strict definition, we would have to take the limit *T* → ∞), and use

(11) $$\delta (f-f^{\prime })S_{xy}(f)=\langle \tilde{x}(f){\tilde{y}}^{*}(f^{\prime })\rangle ,$$

for analytical calculations. A frequency-resolved measure of how well a signal *s*(*t*) can be linearly reconstructed from the spike train *X*(*t*) (or vice versa) is then given by the coherence function

(12) $$C_{sX}(f)=\frac{|S_{sX}(f){|}^{2}}{S_{ss}(f)S_{XX}(f)},$$

which also yields a lower bound on the mutual information rate via the relation (Borst and Theunissen, 1999)

(13) $${ℛ}_{\text{info}}^{s}=-\int_{0}^{f_{c}}df\log_{2}[1-C_{sX}(f)].$$

We can determine the coherence by long simulations of the system and—in the case where the integrate-and-fire model acts as a mostly linear filter—also estimate it analytically. To this end, we consider *X*_in_(*t*), the total *input* to the target neuron *after* it has been weighted by the synaptic dynamics [see Equation (5)]. As detailed in the Appendix, we can express *C*_*s*_*F*_*X*_in__(*f*) and *C*_*s*_*D*_*X*_in__(*f*), the coherences for the two population setup, in terms of single synapse coherences and spectra, all of which have previously been derived in Merkel and Lindner (2010) [see also Rosenbaum et al. (2012) for depressing synapses]. We find

(14) $$C_{s_{F}X_{\text{in}}}(f)=[\frac{1}{N}\frac{1}{C_{s_{F}x_{F}}(f)}\left(1+\frac{S_{x_{D}x_{D}}(f)}{S_{x_{F}x_{F}}(f)}\right)+{\frac{N-1}{N}+\frac{N-1}{N}\frac{|S_{s_{D}x_{D}}(f){|}^{2}}{|S_{s_{F}x_{F}}(f){|}^{2}}]}^{-1}.$$

*C*_*s*_*D*_*X*_in__(*f*) can be obtained from Equation (14) by simply swapping all *F* and *D* subscripts.

## 3. Results

### 3.1. Spectral separation of information

**Figure 2:**
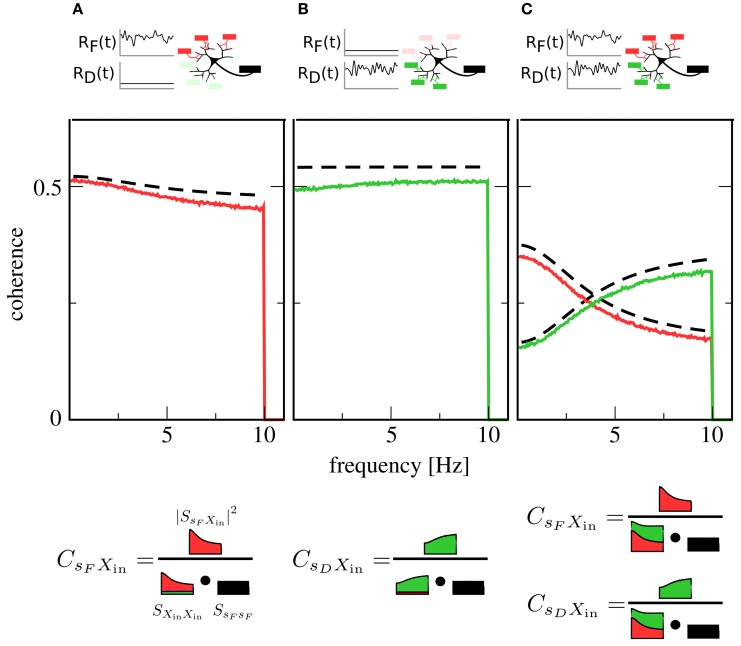
**Coherence between signals and the output spike train *X*(*t*).** Red lines: coherence between *F* signal and *X*(*t*). Green lines: coherence between *D* signal and *X*(*t*). Black dashed lines: theory for *C*_*s*_*F*_*X*_in__(*f*) and *C*_*s*_*D*_*X*_in__(*f*) [see Equation (14)]. In **(A)**, only the *F* population is encoding a signal, while the *D* population is firing at a constant background rate. In **(B)**, the situation is reversed: here, the *D* population is in the signaling state while the *F* population generates background spikes at low rate. In **(C)**, both populations are active and encoding a signal. While in **(A,B)** coherences are rather flat, indicating broadband transmission, the activity of both populations in **(C)** leads to a new effect: Coherence over the *F* signal is more suppressed at low frequencies, while coherence over the *D* signal is more suppressed at high frequencies, leading to a spectral separation of information. A graphical representation of Equation (12) (bottom row) illustrates the role of the input power spectra in shaping this functional dependence. Parameters: *I*_*i*_ = −2.25 nA, ϵ_*F*_ = ϵ_*D*_ = 0.05.

We first examine the case of only one signal. Here, one presynaptic population is active, while the other is firing at a low background rate. This could, for instance, correspond to the presentation of a stimulus to only one sensory modality. Figures 2A,B show plots of the coherence between signal and output spike train in this situation. The coherence can be seen to be mostly flat, i.e., information transfer about the signals shows only a mild frequency dependence [note that for higher cutoff frequencies, the integrate-and-fire neuron itself would induce low-pass filtering (Vilela and Lindner, 2009)].

Simultaneous presence of both signals (and, consequently, activity of both populations) changes this situation qualitatively. As can be seen in Figure 2C, both signals are encoded in the output spike train, but their respective coherence now shows a marked dependence on frequency. While the overall coherence is suppressed for both signals, suppression is stronger at high frequencies than at low frequencies for the *F* signal and vice versa for the *D* signal. In other words, the neuron now preferentially encodes slow components of the *F* signal and fast components of the *D* signal.

To understand the mechanism behind this effect, first consider the situation where only the *F* population is transmitting a signal: spike trains filtered through facilitating synapses have more power at low than at high frequencies (Lindner et al., 2009; Merkel and Lindner, 2010). These spike trains dominate the shape of the total input power spectrum *S*_*X*_in_*X*_in__(*f*), as the *D* population is firing at a low, unmodulated rate. However, the squared cross-spectrum |*S*_*s*_*F*_*X*_in__(*f*)|^2^ has a similar shape, so that most of the frequency dependence cancels out in the calculation of the coherence (cf. Figure 2A, bottom row). This leads to the observed broadband behavior. Such a cancelation, which has first been described in Lindner et al. (2009), also explains the flat coherence in Figure 2B. In contrast, when both *F* and *D* population are transmitting a signal, the cross-spectra are unchanged but the power spectra add up, so that the total power spectrum is rather flat (Figure 2C, bottom row). Consequently, the frequency dependence of the cross spectrum is still apparent in the coherence, leading to the new effect of spectral separation.

Theoretical curves for *C*_*s*_*F*_*X*_in__(*f*) and *C*_*s*_*D*_*X*_in__(*f*) are also shown in Figure 2; while systematically overestimating simulation results, they are in reasonable agreement with them. This is remarkable, as we have only taken synaptic but not neuronal dynamics into account in the derivation of Equation (14). Closer inspection reveals that the theory works as long as the output firing rate is much higher than the cutoff frequency of the signals.

**Figure 3:**
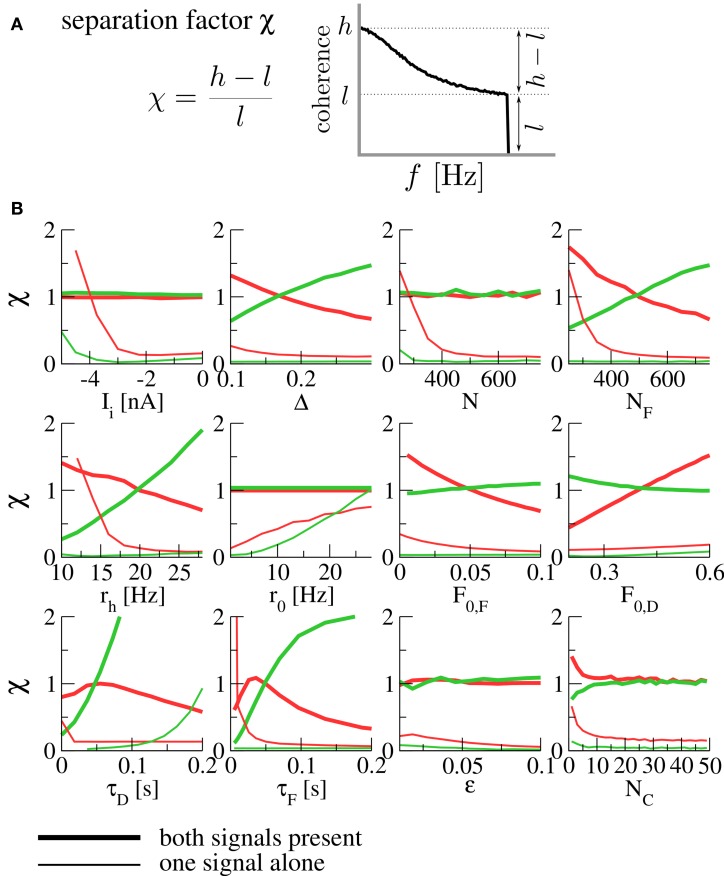
**(A) The separation factor as a measure for the high- or low-pass nature of the coherence. (B) Separation factors under variation of various parameters.** Thick lines denote separation factors obtained when both populations are transmitting a signal, while thin lines mark the case of only one signal (with the other population firing at the background rate *r*_0_). Separation factors for the coherence between the *F* signal and the output spike train are plotted in red, those for the *D* signal in green. For all panels, the fixed parameters are those of Figure 2. *N* is the size of each of the presynaptic populations (meaning that both population sizes were varied at the same time), *N*_*F*_ is the size of the *F* population (with the *D* population fixed at *N*_*D*_ = 500), *r*_*h*_ is the firing rate of a population transmitting a signal (meaning that both *r*_*F*_ and *r*_*D*_ were varied at the same time). For the simulation of varying number of functional contacts *N*_*C*_, the stochastic model of synaptic dynamics was used. When separation factors are low for each signal alone but high in the presence of both signals, we observe the spectral separation effect described above.

In order to quantify the robustness of the spectral separation effect under variation of parameters, we introduce the *separation factor*

(15) $$\chi =\frac{h-l}{l},$$

where

(16) $$h=\underset{0\;\leq f\leq f_{c}}{\max}C_{sX}(f),\;l=\underset{0\;\leq f\leq f_{c}}{\min}C_{sX}(f)$$

are the maximal and minimal values of the coherence function over the signal's frequency band. This is a measure of the high- or low-pass character of the coherence; small values indicate broadband filtering, while large values indicate pronounced high- or low-pass behavior (see Figure 3A). The effect of spectral separation corresponds to a coherence that is rather broadband (low separation factor χ) for each signal alone, but either high- or low-pass when both are present (high separation factor).

In Figure 3B, we plot separation factors for various varied parameters. In general, it can be seen that they are considerably higher when both signals are present than for either of the signals alone for wide ranges of the parameter under variation, confirming the robustness of the effect. As a result of this analysis, we expect the spectral separation effect to be relevant for presynaptic populations of about 400 cells and more, and for synapses with rather short timescales of facilitation and depression [on the order of 100 ms, compatible with the values reported in Lewis and Maler (2002)]. To assess the influence of using a deterministic model, we have run simulations of the stochastic model (which converges to the deterministic model in the limit of infinitely many functional contacts *N*_*C*_). It can be seen in Figure 3B (bottom right) that the same spectral separation effect is observed in the stochastic model already for a relatively modest number of functional contacts (≈ 10).

### 3.2. Stochastic resonance

Until now, we have been concerned with the neuron's information filter properties in a two-signal setup. Another vital issue is whether the second signal increases or decreases the amount of information that is transmitted in total about the first. It is known (Gammaitoni et al., 1998) that additional noise can enhance information transmission in non-linear systems by virtue of stochastic resonance. Basically, additional noise may help the system to cross a threshold; in a neuronal setting, this corresponds to raising the firing rate. Here we inspect in a physiological setting whether a second signal can play a similarly beneficial role as the noise in SR.

**Figure 4:**
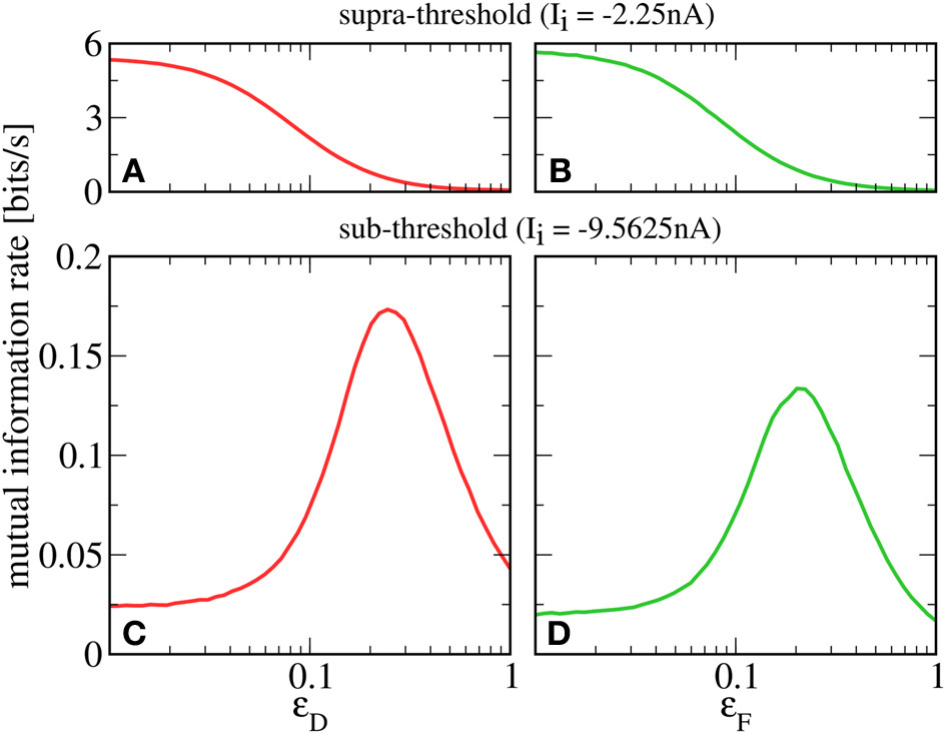
**Mutual information rate (lower bound) between the *F* signal (**A,C**, red) or *D* signal (**B,D**, green), and the output spike train when the modulation amplitude of the respective other signal (ϵ_*D*_ or ϵ_*F*_) is varied.** In the supra-threshold regime (*I*_*i*_ = −2.25 nA; **A,B**), a second signal always impedes the transmission of the first. In contrast, in the sub-threshold regime (*I*_*i*_ = −9.5625 nA; **C,D**), increasing the modulation amplitude of the *D* signal can be beneficial to the transmission of the *F* signal and vice versa. For both regimes, *r*_*F*_ = *r*_*D*_ = 20 Hz; ϵ_*F*_ = 0.05 in **A,C**; ϵ_*D*_ = 0.05 in **B,D**.

A prerequisite for SR is that the neuron is in the sub-threshold regime (reached in our model by lowering *I*_*i*_), in which most cortical neurons seem to operate (Shadlen and Newsome, 1998). For this regime, we plot in Figures 4C,D a lower bound for the mutual information rate between the *F* or *D* signal and the output spike train [${ℛ}_{\text{info}}^{s}$, Equation (13)] as a function of the second signal's amplitude ϵ_*D*_ or ϵ_*F*_ (with *r*_*F*_ = *r*_*D*_ = 20 Hz). It can be seen that, up to some optimal value, a stronger *D* signal indeed helps the transmission of the *F* signal and vice versa—a clear-cut case of SR. This is in contrast to the supra-threshold regime considered in the previous section, where adding a second signal always impedes the transmission of the first (see Figures 4A,B).

It is instructive to compare our results to other scenarios of SR. Two prominent sources of neuronal noise lie in the stochasticity of spike arrival times (*synaptic noise*) and the stochastic opening and closing of ion channels (*channel noise*). Both synaptic noise (Rudolph and Destexhe, 2001; Torres et al., 2011) as well as channel noise (Schmid et al., 2001) can give rise to SR. Synaptic noise is already present in our model and can be controlled by the baseline rate of presynaptic populations (a higher rate leads to a higher mean input as well as larger fluctuations around this mean). As a caricature of fast channel noise, we add Gaussian white noise $\sqrt{2D}\xi (t)$ to the r.h.s. of Equation (7); it is this kind of noise that was used in most previous studies of SR. In the following, we consider information transmission about the *D* signal when the amplitude of the *F* signal is varied; qualitative results and conclusions drawn from them are the same in the inverse case.

**Figure 5:**
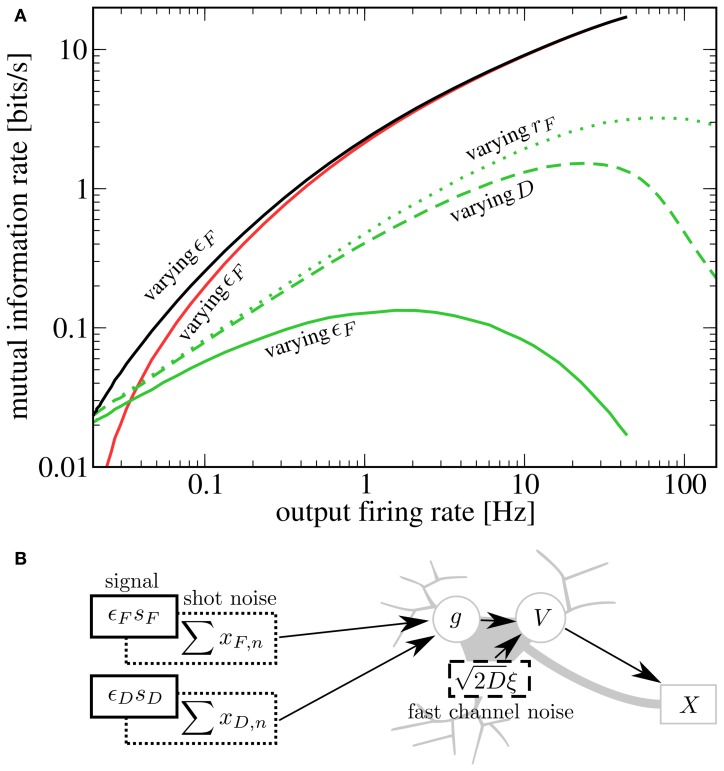
**(A) Comparison of different kinds of stochastic resonance.** Starting from *I*_*i*_ = −9.5625 nA, *r*_*F*_ = *r*_*D*_ = 20 Hz, ϵ_*D*_ = 0.05, ϵ_*F*_ = 0, *D* = 0 nA^2^s, we increase either ϵ_*F*_ (up to 1), *r*_*F*_ (up to 60 Hz) or *D* (up to 0.14 nA^2^s). For each case, we plot ${ℛ}_{\text{info}}^{s_{D}}$ as a function of the output firing rate (green lines; solid, dashed, and dotted for varying ϵ_*F*_, *D*, and *r*_*F*_, respectively). Additionally, we plot ${ℛ}_{\text{info}}^{s_{F}}$ (red line) and ${ℛ}_{\text{info}}^{s_{F}}+{ℛ}_{\text{info}}^{s_{D}}$ (black line) for the case of varying ϵ_*F*_. A second signal is less effective at enhancing information transmission of the first signal than either channel noise or an increase in presynaptic-synaptic firing. However, as the helpful signal is transmitted as well, the total rate of information transmission is highest in this case. **(B)** Schematic depiction of the sources of noise in the system. In addition to the fluctuating signals, noise is introduced by the stochastic firing of the presynaptic-synaptic populations and the stochasticity of ion channels.

To compare the effects of the different noise sources on signal transmission, we start from *r*_*F*_ = 20 Hz, *D* = 0 nA^2^s, ϵ_*F*_ = 0 and increase either the noise strength, the baseline rate, or the modulation amplitude. We see in Figure 5A that for SR via a second signal (solid green line), the information rate about the *D* signal at a given output firing rate is lower than for SR via channel noise (dashed green line). This can be understood by considering that the second signal, which plays the role of the noise, has power in exactly the same frequency range as the one that we want to transmit. It is plausible that with weaker but more broadband noise (e.g., white noise), one can achieve the same output firing rates (with help from the high frequency components) with a lower contamination of the relevant frequency range. This reasoning is consistent with the experimental finding that white noise is more “effective” for SR than low-frequency noise (Nozaki et al., 1999). Both the second signal as well as white noise yield lower peak mutual information rates than synaptic noise (green dotted line). This is due to the increase in mean input that goes along with an increase in presynaptic firing rate (raising the output firing rate without introducing noise). Although in our scenario, the information rate about the *D* signal is smaller than in the other two cases, there is a clear advantage of enhancing the transmission of one signal by adding another signal: both add meaningful information to the output spike train. Indeed, the total rate of information transmission for two signals (black line) exceeds that in all the other cases. At least in the case of channel noise, we can be certain that considering the total rate of information would not be meaningful, because of the molecular (not signal-related) origin of these fluctuations.

**Figure 6:**
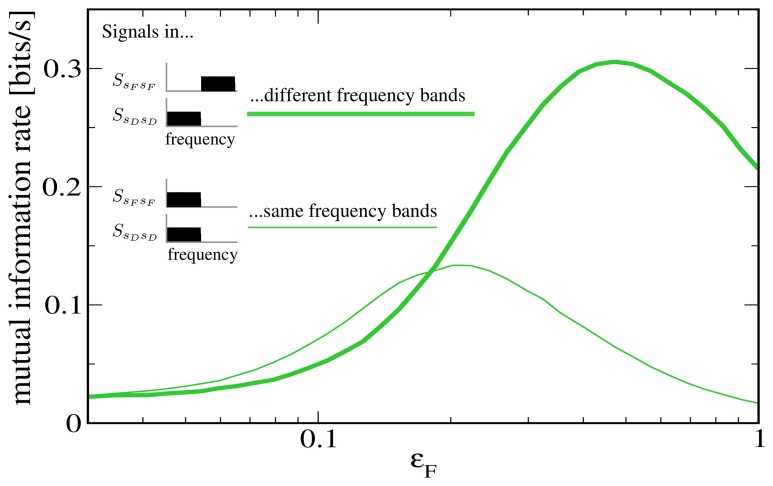
**Mutual information rate (lower bound) between the *D* signal and the output spike train when the modulation amplitude ϵ_*F*_ is varied, both for the case considered above (both signals in the same frequency range) and for the case where the *F* signal has been shifted to values between 10 and 20 Hz.** The shifted signal is clearly more effective at enhancing information transmission about the *D* signal. Other parameters as in Figures 4, 5.

To further illustrate that the reason for the low rate of information transmission in the case of signal-meditated SR in Figure 5 is mainly the overlap of both signals in frequency space, we plot in Figure 6 a comparison to a “detuned” setup, i.e., one in which the *F* signal has been shifted to frequencies between 10 Hz and 20 Hz. It can be seen that this shifted version of the *F* signal is indeed considerably more effective at increasing information transmission about the *D* signal.

### 3.3. Comparison to a setup with static synapses

The filtering effect described in section 3.1 is clearly a consequence of heterogeneous synaptic short-term plasticity; it would not occur in a setup with static synapses. In contrast, one can expect the effect of signal-mediated SR (section 3.2) to occur independently of synaptic dynamics, as the primary beneficial effect of the second signal is an increase in postsynaptic firing rate, something that can be achieved with static synapses as well. In the following, we explicitly compare our setup to one with static synapses.

**Figure 7:**
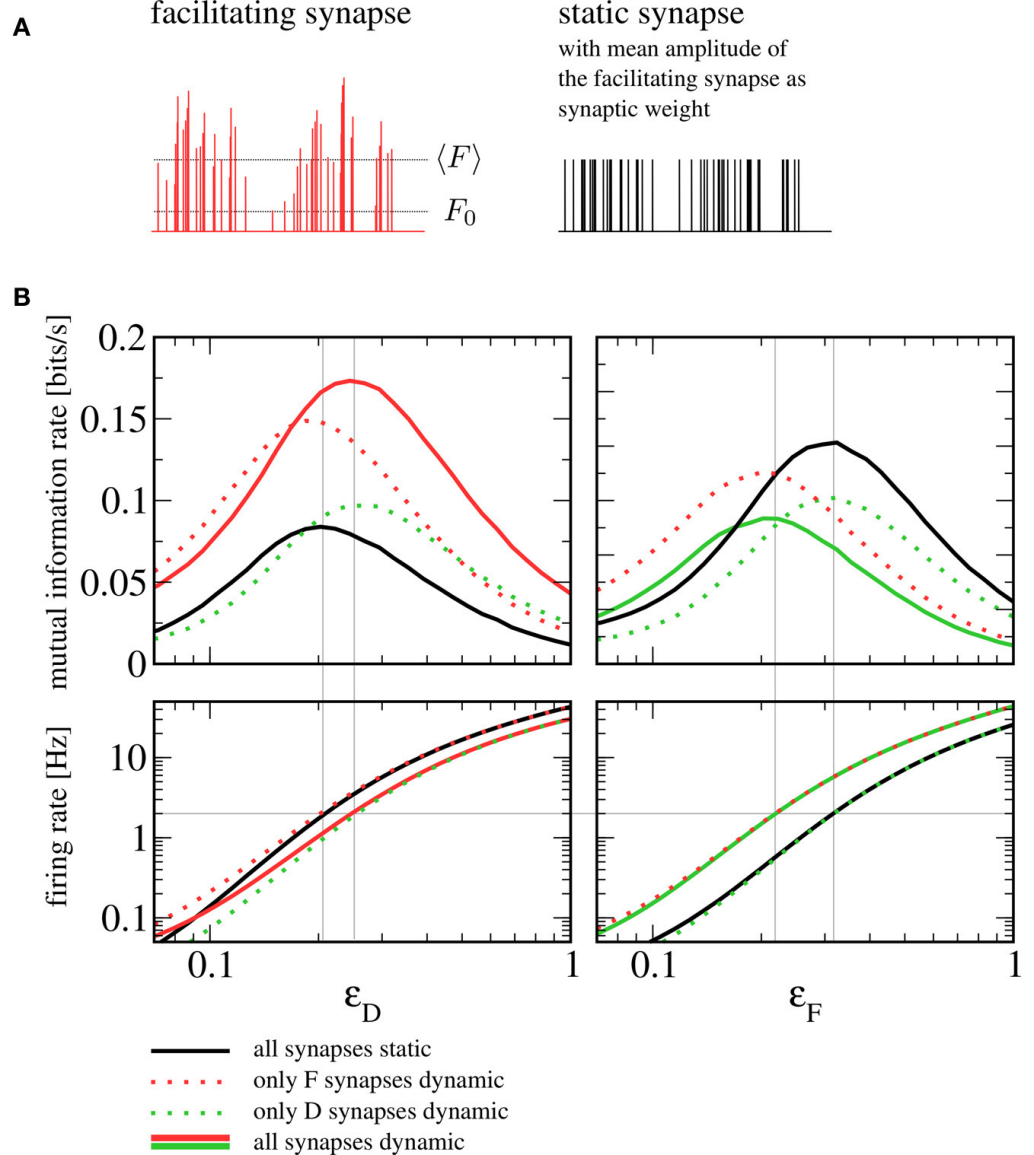
**Comparison to a setup with static synapses. (A)** Schematic depiction of a spike train's amplitudes after passing through a facilitating synapse (with the mean amplitude 〈*F*〉 and the intrinsic release probability *F*_0_ indicated by dotted lines) and the same spike train with static amplitudes chosen to equal the mean amplitude of the dynamic synapse. **(B)** Mutual information rates (lower bound) over *F* signal (left) and *D* signal (right) when the amplitude of the respective other signal is varied (sub-threshold regime, *I*_*i*_ = −9.5625 nA). The solid red and green lines denote information rates for the setup with heterogeneous short-term plasticity (as shown in Figure 4), the dotted and the solid black lines correspond to setups in which one or both synaptic populations have been replaced by static synapses. It can be seen that replacing dynamic by static synapses does not qualitatively change information transmission rates. In the bottom row, we plot the firing rate of the postsynaptic cell. If the signal that plays the role of the beneficial noise is strong, the firing rate can be seen to depend only on the type of synapses (static or dynamic) through which this signal enters. The gray horizontal line indicates a firing rate of 2 Hz. Gray vertical lines indicate values of ϵ_*D*_ and ϵ_*F*_ at which this firing rate is attained; they can be seen to be in good agreement with the position of the peaks in the mutual information rate.

How should one choose the amplitude of static synapses to allow for a meaningful comparison? An obvious approach would be to use the intrinsic release probabilities *F*_0, *F*_ and *F*_0, *D*_ as the respective weights, which would correspond to taking the dynamic synapses and switching off short-term plasticity ( for example by taking the limit of infinitely fast recovery). However, the most prominent difference would then lie in the mean amplitude of the postsynaptic spike trains. In other words, shutting off facilitation (depression) would lead to a dramatic reduction (increase) in the rate of vesicle release. Instead of assessing the influence of synaptic dynamics on information transmission, we would essentially be comparing strong to weak synapses, with the obvious outcome. Here we choose the amplitudes of static synapses to equal the mean of those in the dynamic case (see Figure 7A). Note that more sophisticated schemes exist to tune parameters of the conductance dynamics such that not only the mean but also the variance of the conductance are the same for static and dynamic synapses (Lindner et al., 2009).

In Figure 7B, we show a comparison to a setup where some or all dynamic synapses have been replaced by static ones. Generally, the mutual information rate shows the same qualitative behavior for all variants. In particular, the SR peaks persist when one or both synaptic populations are replaced by static synapses. For our standard sets of parameters, the peak for all-static synapses (black lines) is shifted somewhat to the left for the *F* signal and somewhat to the right for the *D* signal; using static synapses can be seen to yield lower mutual information rates for the *F* signal and higher mutual information rates for the *D* signal. As we detail below, the shift can be understood from the change of character (dynamic to static) of the synapses through which the beneficial “noise” signal enters, in the following referred to as “noise synapses”. The increase or reduction in information transmission rate, on the other hand, can be traced back to the change of the signal synapses.

Let us first discuss the shift in the noise signal amplitudes ϵ_*D*_ and ϵ_*F*_ (the abscissae in Figure 7B, left and right, respectively) that maximize the information transmission in the sub-threshold case (top panels in Figure 7B). To understand these shifts, it is instructive to look at the postsynaptic firing rates (Figure 7B, bottom row). The beneficial effect of noise in SR is based on a rapid increase in firing rate that this noise brings about. It can be seen that for the particular parameters we have chosen, a unique condition for a maximized information transfer is that the postsynaptic firing rate is about 2 Hz. The shift in the maximum can be thus understood by addressing the much simpler question what sets the output firing rate in the different combinations of static and dynamic synapses. In the range where the maximum is attained, the rate is mainly determined by the nature of the noise synapses. Hence, we find that the maxima of the mutual information rate are attained at the same level if noise synapses are dynamic (solid red and dotted green in the left panel, solid green and dotted red in the right panel) or if they are static (dotted red and solid black in the left panel; dotted green and solid black in the right panel). For the same mean amplitude of the postsynaptic input, the facilitating (depressing) synapse introduces more (less) power in the relevant low-frequency range than the static synapse does. In order to achieve the same firing rate of 2 Hz with a static synapse, we thus have to increase (decrease) the noise signal amplitude compared to the situation with a facilitating (depressing) synapse.

Finally, we would like to address the question why with the chosen parameters facilitating (depressing) synapses seem to transfer more (less) information about the stimulus than static ones do. First of all, closer inspection of the theoretical formula [Equation (14)] indicates that this does not have to be the case for all parameter sets. We can, however, make plausible that we expect a larger (smaller) information rate for facilitating (depressing) synapses compared to static synapses for a large number of synapses and a moderate-to-large amplitude of the noise signal. If we have a large amount of noise in the system that is independent of the channel that carries the signal, the difference between dynamic and static signal synapses arises due to the differences between input-signal-output-spike-train cross-spectra. Facilitating synapses have a higher cross-correlation with the signal because the amplitudes of the spikes change in parallel with the rate modulation of input spikes and this higher cross-correlation leads to a higher coherence. Depressing synapses, on the contrary, change the amplitude of input spikes in an opposite sense to the rate modulation and thus have a smaller cross-correlation between rate modulation and output spikes, thus a lower coherence.

## 4. Discussion

How a single time-varying signal is encoded in the neural spike train has been the subject of numerous studies [see e.g., Borst and Theunissen (1999) and references therein]. In this paper, we have demonstrated two distinct effects that arise in a setup with two signals. Firstly, adding a second signal can switch the neuron's information filter properties from broadband to frequency selective, if the two signals impinge on synapses that display opposite kinds of synaptic short-term plasticity. Secondly, the second signal can increase the respective rate of information transmission for both signals, when the neuron is in the fluctuation-driven regime. Although we have demonstrated these effects in the same model setup, it should be noted that they are distinct in nature and that the latter does not depend on synaptic plasticity.

We have explicitly compared our setup to one in which one or both synaptic populations have been replaced by static synapses and found that the influence of one signal on the total rate of information transmission about the other signal does not depend on synaptic plasticity in a qualitative way. In particular, the SR effect is also observed with static synapses. It is noteworthy that facilitating synapses yield higher mutual information rates than static synapses with the same mean amplitude, while the opposite is true when comparing depressing and static synapses. This is non-trivial, as the coherence for a single static synapse is always higher than for a single facilitating or depressing synapse (Merkel and Lindner, 2010).

While the situation that a neuron receives more than one signal arises naturally in the context of multisensory integration, it is most likely more general. One of the signals might, for instance, represent an actual sensory stimulus that is passed through a feed-forward structure (bottom–up), while the other may be an internally generated top–down signal from higher cortical areas. In our setup, such an internal signal could control the transmission of the stimulus, either by inducing a filtering of information, or by enhancing transmission through SR. Note that in this general case, the requirement of having independent signals might need to be relaxed.

The spectral separation effect can be considered a non-trivial interaction between the two signals. In the presence of both signals, the neuron acts as an information filter—its coherence function deviates from a flat form, indicating that some frequency components get transmitted more reliably than others. In general, such a frequency dependence of the coherence can result from non-linearity in the system or from temporal structure in the inputs (colored noise). In our setup, temporal correlations in the inputs are induced by facilitation or depression, and, as we have shown, they are already enough to understand the separation effect: When entering through a homogeneous synaptic population, noise and signal are filtered in a similar way, so that the signal-to-noise ratio stays almost constant and information transmission is largely frequency independent (Lindner et al., 2009; Merkel and Lindner, 2010). With respect to the transmission of this signal, a second signal acts as additional noise, and if it is filtered differently, the frequency dependence no longer cancels. With synaptic populations of opposite kinds, this effect is especially pronounced, as the filtered second signal adds more power at those frequencies that are already suppressed by the first filter.

The spectral separation effect is most certainly not the only way frequency-selective information transmission could be implemented. As demonstrated by Rosenbaum et al. (2012), depressing synapses alone can already constitute a high-pass information filter, due to the stochasticity in vesicle uptake and release. We have run simulations with stochastic synapses, and found that in the setting we consider, stochasticity-induced filtering seems to be negligible, even when the number of functional contacts is small. However, in a situation with smaller pre-synaptic populations or long time scales of vesicle recovery τ_*d*_, they should become relevant.

As shown, we have verified that the information spectral separation effect persists when parameters are varied withing reasonable bounds. Furthermore, because the effect is a result of correlations induced by synaptic dynamics rather than of non-linearity in the system (such as the spike-generating mechanism) or a network mechanism (Middleton et al., 2009; Sharafi et al., 2013), similar results can be expected with neuron models that are more realistic than the integrate-and-fire model we used. It should be noted that our approach contains implicit assumptions (most importantly stationarity, coding of information in the instantaneous firing rate and Poissonian input statistics) that are probably not always justified. Relaxing these assumptions is an interesting task for future studies.

The functional role of the information filtering effect at the level of signal processing networks is still unclear and merits further investigation. At the single cell level, our findings have general implications for the study of filtering properties of synapses: It may make more sense to consider filtering on the neuronal level (taking potentially inhomogeneous synaptic populations into account) than to discuss the filtering properties of a certain kind of synapse in isolation.

The beneficial effect that one signal can have on the transmission of the other is a manifestation of SR, which, loosely speaking, refers to the enhancement of signal transmission by a non-vanishing amount of noise. In our case, the place of this noise is taken by the second signal, which does not enter the neuron directly, but as a modulation of presynaptic firing rates. We have compared this variant of SR to others previously discussed in the literature (Rudolph and Destexhe, 2001; Schmid et al., 2001; Torres et al., 2011) and found a second signal to be less effective in enhancing information transmission (about the first signal) than traditionally considered noise sources. However, an obvious benefit of this scenario is that both signals contain information that is transmitted by the neuron.

It has been noted before that “the input signal for one computation may well be considered noise for a different computation” (McDonnell and Ward, 2011) and that variability may be “signal, even though it would look like noise” (Masquelier, 2013), but the present work is to our knowledge the first to explore such a scenario explicitly. It is an attractive idea that neural systems might exploit SR not by adding noise that serves no other purpose, but rather through the interplay of signals that are being processed anyway.

### Conflict of interest statement

The authors declare that the research was conducted in the absence of any commercial or financial relationships that could be construed as a potential conflict of interest.

## Appendix

### Derivation of the coherence formula for the two-signal setup

Our aim is to express the coherence in the two-signal setup,

(A1) $$C_{sX_{\text{in}}}(f)=\frac{|S_{sX_{\text{in}}}(f){|}^{2}}{S_{ss}(f)S_{X_{\text{in}}\;X_{\text{in}}}(f)},$$

with *s* = *s*_*F*_, *s*_*D*_, in terms of the single-synapse cross- and power-spectra *S*_*s*_*F*_*x*_*F*__(*f*), *S*_*x*_*F*_*x*_*F*__(*f*), *S*_*s*_*D*_*x*_*D*__(*f*) and *S*_*x*_*D*_*x*_*D*__(*f*), where *S*_*s*_*F*_*x*_*F*__(*f*) is the cross spectrum between the *F* signal and a spike train that has been weighted by a single facilitating synapse, *S*_*x*_*F*_*x*_*F*__(*f*) is the power spectrum for such a spike train, and *S*_*s*_*D*_*x*_*D*__(*f*) and *S*_*x*_*D*_*x*_*D*__(*f*) are the corresponding quantities for a depressing synapse. They have been derived in Merkel and Lindner (2010) and read

(A2) $$S_{s_{F}x_{F}}(f)=\frac{r_{F}\epsilon_{F}}{2f_{c}}\left(F_{0,l}+\Delta_{l}r_{F}\tau_{F}\left(1+\frac{1}{1-2\pi if\tau_{F}}\right)\right),$$

(A3) $$S_{x_{F}x_{F}}(f)=r_{F}\frac{{\left(F_{1,\;l}+\Delta_{l}r_{F}\tau_{F}\right)}^{2}+{\left(2\pi f\tau_{F}\right)}^{2}F_{1,\;l}^{2}}{1+{\left(2\pi f\tau_{D}\right)}^{2}}+\frac{1}{2}\Delta_{l}^{2}r_{F}\tau_{f},$$

(A4) $$S_{s_{D}x_{D}}(f)=F_{0,D}\frac{r_{D}\epsilon_{D}}{2f_{c}}\left(1-\frac{F_{0,D}r_{D}\tau_{D}/\beta_{D}}{1-2\pi if\tau_{D}/\beta_{D}}\right),$$

(A5) $$S_{x_{D}x_{D}}(f)=\frac{F_{0,\;D}^{2}}{\beta_{D}^{3}(1+F_{0,\;D}r_{D}\tau_{D}-F_{0,D}^{2}r_{D}\tau_{D}/2)}\times \frac{1+{\left(2\pi f\tau_{D}\right)}^{2}}{1+{\left(2\pi f\tau_{D}/\beta_{D}\right)}^{2}},$$

where

(A6) $$F_{0,\;l}:=F_{0,\;F}+\frac{{\left(\Delta r_{F}\tau_{F}\right)}^{2}\left(1-F_{0,\;F}\right)}{\gamma^{2}}+\frac{\Delta^{2}r_{F}\tau_{F}{\left(1-F_{0,\;F}\right)}^{2}}{6\gamma^{3}}\left(1-\frac{\Delta \left(1+9r_{F}\tau_{F}\right)}{\gamma }\right),$$

(A7) $$\Delta_{l}:=\frac{\Delta {\left(1-F_{0,F}\right)}^{2}}{\gamma^{2}}\cdot \left(1-\frac{2\Delta }{3\gamma }+\frac{\Delta^{2}\left(1+3r_{F}\tau_{F}\right)}{2\gamma^{2}}\right),$$

(A8) $$\gamma :=1-F_{0,F}+\Delta r_{F}\tau_{F},$$

(A9) $$F_{1,\;l}:=F_{0,\;l}+\Delta_{l}r_{F}\tau_{F},$$

(A10) $$\beta_{D}:=1+F_{0,D}r_{D}\tau_{D}.$$

In the following, we derive the coherence between the *F* signal and the total weighted input *X*_in_(*t*). The derivation for the *D* signal is completely analogous and can be obtained by swapping all *F* and *D* subscripts. We begin with the cross spectrum between *s*_*F*_(*t*) and *X*_in_(*t*). Using Equations (11) and (5), the independence between the *F* signal and the spike trains entering through the *D* synapses, the stationarity of signals and spike trains, and the uniformity of *F* synapses, we obtain

(A11) $$\delta (f-f^{\prime })S_{s_{F}\;X_{\text{in}}}(f)$$

(A12) $$=\;{\langle {\tilde{s}}_{F}(f){\tilde{X}}_{I}^{*}(f^{\prime })\rangle }_{\xi ,s}$$

(A13) $$={\langle {\tilde{s}}_{F}(f)\left(\sum_{n}^{N}{\tilde{x}}_{F,\;n}^{*}(f^{\prime })+\sum_{k}^{N}{\tilde{x}}_{D,k}^{*}(f^{\prime })\right)\rangle }_{\xi ,s}$$

(A14) $$=\sum_{n}^{N}{\langle {\tilde{s}}_{F}(f){\tilde{x}}_{F,n}^{*}(f^{\prime })\rangle }_{\xi ,s}+\sum_{n}^{N}{\langle {\tilde{s}}_{F}(f)\rangle }_{s}{\langle {\tilde{x}}_{D,k}^{*}(f^{\prime })\rangle }_{\xi }$$

(A15) $$=N{\langle {\tilde{s}}_{F}(f){\tilde{x}}_{F}^{*}(f^{\prime })\rangle }_{\xi ,s},$$

where 〈.〉_*s*_ denotes averaging over the appropriate stimulus ensemble(s), 〈.〉_ξ_ averaging over the ensemble(s) of (inhomogeneous Poisson) spike trains and 〈.〉_ξ_, _*s*_ averaging over all ensembles. Thus, we have

(A16) $$\begin{array}{l} S_{s_{F}\;X_{\text{in}}}(f)=NS_{s_{F}x_{F}}(f). \end{array}$$

In the derivation of Equations (A2–A5), it has been assumed that signals are weak, so that linear response theory,

(A17) $$\langle {\tilde{x}}_{F}(f)=\chi_{F}(f){\tilde{s}}_{F}(f)\rangle ,$$

can be applied. Here, χ_*F*_(*f*) is the susceptibility of facilitating synapses. It is implicitly given by Equation (A2), which can be written as

(A18) $$S_{s_{F}x_{F}}(f)=\chi_{F}^{*}(f)S_{s_{F}s_{F}}(f).$$

Note that we have assumed the two signals to have identical statistics and unit variance, so that *S*_*s*_*F*_*s*_*F*__(*f*) = *S*_*s*_*D*_*s*_*D*__(*f*) = 1/(2*f*_*c*_).

Exploiting the independence between spike trains entering via *F* and *D* synapses, we obtain for the power spectrum

(A19) $$\delta (f-f^{\prime })S_{X_{\text{in}}\;X_{\text{in}}}(f)$$

(A20) $$={\langle {\tilde{X}}_{I}(f){\tilde{X}}_{I}^{*}(f^{\prime })\rangle }_{\xi ,s}$$

(A21) $$=\sum_{i,j}^{N}{\langle {\tilde{x}}_{F,\;i}(f){\tilde{x}}_{F,j}^{*}(f^{\prime })\rangle }_{\xi ,s}+\sum_{i,l}^{N}{\langle {\tilde{x}}_{D,\;i}(f){\tilde{x}}_{D,l}^{*}(f^{\prime })\rangle }_{\xi ,s},$$

and, because

(A22) $${\langle {\tilde{x}}_{F,\;i}(f){\tilde{x}}_{F,j}^{*}(f^{\prime })\rangle }_{\xi ,s}={\langle {\tilde{x}}_{F,\;i}(f){\tilde{x}}_{F,j}^{*}(f^{\prime })\rangle }_{\xi ,s}\delta_{ij}+{\langle {\langle {\tilde{x}}_{F,i}(f)\rangle }_{\xi }{\langle {\tilde{x}}_{F,j}(f^{\prime })\rangle }^{*}{}_{\xi }\rangle }_{{}_{\text{s}}}$$

(A23) $$={\langle {\tilde{x}}_{F,\;i}(f){\tilde{x}}_{F,j}^{*}(f^{\prime })\rangle }_{\xi }\delta_{ij}+{\left|\chi_{F}(f)\right|}^{2}{\langle {\tilde{s}}_{F}(f){\tilde{s}}_{F}^{*}(f^{\prime })\rangle }_{s}$$

(and likewise for the depressing synapses), the power spectrum can be written as

(A24) $$S_{X_{\text{in}}\;X_{\text{in}}}(f)=N(S_{x_{F}x_{F}}(f)+S_{x_{D}x_{D}}(f))+N(N-1)\times ({\left|\chi_{F}(f)\right|}^{2}S_{s_{F}s_{F}}(f)+{\left|\chi_{D}(f)\right|}^{2}S_{s_{D}s_{D}}(f)).$$

Inserting Equation (A15) and (A24) into the definition of the coherence (Equation A1) and using Equation (A18), we finally obtain

(A25) $$C_{s_{F}X_{\text{in}}}(f)=[\frac{1}{N}\frac{1}{C_{s_{F}x_{F}}(f)}\left(1+\frac{S_{x_{D}x_{D}}(f)}{S_{x_{F}x_{F}}(f)}\right)+{\frac{N-1}{N}+\frac{N-1}{N}\frac{|S_{s_{D}x_{D}}(f){|}^{2}}{|S_{s_{F}x_{F}}(f){|}^{2}}]}^{-1},$$

where *C*_*s*_*F*_*x*_*F*__(*f*) is the single synapse coherence.
